# MST3 promotes proliferation and tumorigenicity through the VAV2/Rac1 signal axis in breast cancer

**DOI:** 10.18632/oncotarget.7542

**Published:** 2016-02-20

**Authors:** Chien-Yu Cho, Kuo-Ting Lee, Wei-Ching Chen, Chih-Yang Wang, Yung-Sheng Chang, Hau-Lun Huang, Hui-Ping Hsu, Meng-Chi Yen, Ming-Zong Lai, Ming-Derg Lai

**Affiliations:** ^1^ Department of Biochemistry and Molecular Biology, National Cheng Kung University, Tainan, Taiwan, ROC; ^2^ Institute of Basic Medical Sciences, National Cheng Kung University, Tainan, Taiwan, ROC; ^3^ Department of Surgery, National Cheng Kung University, Tainan, Taiwan, ROC; ^4^ Center for Infectious Diseases and Signaling Research, College of Medicine, National Cheng Kung University, Tainan, Taiwan, ROC; ^5^ Department of Emergency Medicine, Kaohsiung Medical University Hospital, Kaohsiung Medical University, Kaohsiung, Taiwan, ROC; ^6^ Institute of Molecular Biology, Academia Sinica, Taipei, Taiwan, ROC; ^7^ Graduate Institute of Immunology, National Taiwan University, Taipei, Taiwan, ROC

**Keywords:** MST3, VAV2, Rac1, cyclin D1, breast cancer

## Abstract

MST3 (mammalian STE20-like kinase 3) belongs to the Ste20 serine/threonine protein kinase family. The role of MST3 in tumor growth is less studied; therefore, we investigates the function of MST3 in breast cancer. Here, we demonstrate that MST3 is overexpressed in human breast tumors. Online Kaplan-Meier plotter analysis reveals that overexpression of MST3 predicts poor prognosis in breast cancer patients. Knockdown of MST3 with shRNA inhibits proliferation and anchorage-independent growth *in vitro*. Downregulation of MST3 in triple-negative MDA-MB-231 and MDA-MB-468 breast cancer cells decreases tumor formation in NOD/SCID mice. MST3 interacts with VAV2, but not VAV3, as demonstrated by co-immunoprecipitation and confocal microscopy. By domain mapping of MST3, we determine that the proline-rich region of MST3 (^353^KDIPKRP^359^) interacts with the SH3 domain of VAV2. Mutation of the two proline residues in this domain significantly attenuates the interaction between MST3 and VAV2. Overexpression of wild-type MST3 (WT-MST3), but not proline-rich-deleted MST3 (ΔP-MST3), enhances the proliferation rate and anchorage-independent growth of MDA-MB-468 cells. Overexpression of MST3 increases VAV2 phosphorylation and GTP-Rac1, whereas downregulation of MST3 or delivery of ΔP-MST3 results in a reduction of VAV2 and Rac1 activation. Knockdown of MST3 inhibits cyclin D1 protein expression. The Rac1 inhibitor EHop-016 attenuates cell proliferation induced by WT-MST3. Finally, Knockdown of MST3 or Rac1 inhibitor decreases cyclin D protein expression, which is important for tumor growth. These results indicate that MST3 interacts with VAV2 to activate Rac1 and promote the tumorigenicity of breast cancer.

## INTRODUCTION

Ste20 kinase, which is a classic mitogen-activated protein kinase kinase kinase kinase (MAP4K), regulates a wide range of fundamental cellular processes such as the cell cycle, apoptosis, development, growth and stress responses [1-2]. Based on structural relationships, the mammalian Ste20 kinase family comprises the P21-activated kinase (PAK) subfamily and germinal center kinase (GCK) subfamily. The MST (mammalian Ste20-like) protein kinases, MST1, -2, -3, and -4, are members of the GCK family. Based on the similarity between the sequences inside and outside their kinase domains, the MST family kinases can be further divided into the GCK-II subgroup (MST1 and MST2) and GCK-III subgroup (MST3 and MST4) [3].

MST3 is cleaved and translocated into the nucleus during apoptosis. Caspase-3 cleaves MST3 at AETD^313^, which is the junction of the N-terminal kinase domain and the C-terminal regulatory domain [4]. MST3 contains a nuclear localization sequence (NLS) at the C-terminus of its kinase domain (residues 278-292) and a nuclear export signal (NES) in the regions of amino acids 335-386 [5]. Myristoylation of the cleaved C-terminal MST3 may be important for its proper localization [6]. MST3 is also involved in the caspase-independent apoptotic pathway in response to staurosporine [7]. On the other hand, MST3 may act upstream of the nuclear Dbf2-related kinase (NDR) to control cell cycle and growth. NDR, a serine/threonine protein kinase, directly phosphorylates the cyclin-Cdk inhibitor protein p21 at S146, reducing its stability. In addition, NDR is phosphorylated by MST3 at Thr442 to enhance cell cycle progression [8-9]. MST3 directly phosphorylates and inactivates protein tyrosine phosphatase PTP-PEST, which enhances cell migration by enhancing the tyrosine phosphorylation of paxillin Y31 and Y118 [10]. In the mammalian central nervous system, MST3 phosphorylates TAO1/2 kinase at T440/T475, which mediates Myosin Va function to enhance synapse development [11].

MST kinase can be activated through autophosphorylation. Autophosphorylation of threonine residues is essential for MST1-3 kinase activity, and the mutation of T183-MST1/T180-MST2/T178-MST3 to alanine eliminates its kinase activity [10, 12-13]. Alteration of threonine 178 of MST3 to glutamic acid, which mimics the phosphorylation state, enhances MST3 kinase activity. MST3 is phosphorylated at threonine 328 upon stimulation by calyculin A, a serine/threonine phosphatase 1/2A inhibitor, but this phosphorylation is not necessary for MST3 activity. The phosphorylation of T328-MST3 is suggested to create a docking region, which is required for the association between MST3 and the MO25 scaffolding protein [14]. Phosphorylation of serine 79 on MST3 by cyclin-dependent kinase 5 (CDK5) regulates neuronal migration through Rho A-dependent actin dynamics [15].

MST4 kinase activity is stimulated significantly by epidermal growth factor receptor (EGFR) ligands, which are known to promote the growth of prostate cancer cells. [16]. MST4 also plays an oncogenic role in human pituitary tumors that responds to a hypoxic microenvironment to promote tumorigenesis [17]. MST3 phosphorylates NDR and induces the NDR signaling pathway that enhances cell cycle progression and cell growth. In addition, MST3 can phosphorylate PTP-PEST and inhibit the tyrosine phosphatase activity of PTP-PEST. PTP-PEST has been identified as a tumor suppressor by interacting with oncogenic tyrosine kinase [18]. Recently, MST3 was shown to mediate the regulation of the contractile actomyosin machinery by FAM40A, a component of the STRIPAK complex, and is required in metastasis of breast cancer [19].

The VAV family serves as a guanine nucleotide exchange factor (GEF) for the Rho family of GTPases [20]. VAV proteins contain protein subdomains, including an NH_2_-terminal calponin homology (CH) domain, an acidic domain (AD), a Dbl homology (DH) domain followed by a pleckstrin homology (PH) domain, a cysteine-rich region, an Src-homology 2 (SH2) domain, and two Src-homology 3 (SH3) domains. The VAV family includes VAV1, VAV2, and VAV3. VAV1 is expressed primarily in cells of the hematopoietic system, whereas VAV2 and VAV3 have broader patterns of expression [21-22]. All three VAV family members can induce the transformation of NIH3T3 cells when overexpressed as truncated constitutively active proteins [23-25]. VAV1 is expressed in pancreatic adenocarcinoma [26], neuroblastoma [27], and melanoma [28] and overexpressed in B-cell chronic lymphocytic leukemia [29]. VAV3 is overexpressed in prostate cancer and leukemia during progression [30-33]. VAV2 is important for tumor growth, neo-angiogenesis and metastasis in several cancers [34-38]. VAV2 and VAV3 are also required for skin tumor initiation and promotion [39].

Signaling study of MST3 has revealed that MST3 may be oncogenic; however, the oncogenic role of MST3 has not been studied in human cancers. Therefore, we aimed to investigate the function of MST3 in breast cancer. In addition, a systematic screening for VAV2 interaction using a yeast two-hybrid approach suggested that MST3 may be a VAV2 partner, but the interaction was not determined in mammalian cells [40]. In addition, the biologic function of the possible interaction between MST3 and VAV2 has not been studied yet. In this report, we demonstrate that MST3 is overexpressed in human breast cancer and predicts poor prognosis. Furthermore, VAV2 is essential for the oncogenic activity of MST3 in breast cancer.

## RESULTS

### MST3 is overexpressed in breast cancer tissue and predicts patient survival

**Figure 1:**
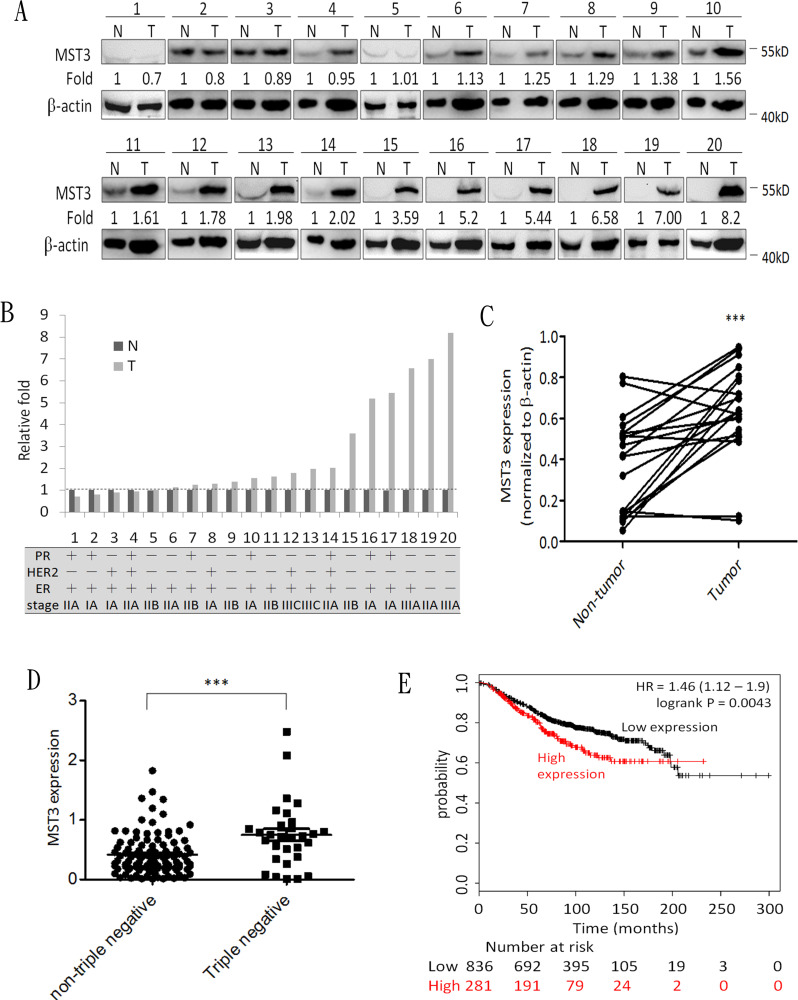
MST3 is up-regulated in breast cancer tissue, and high expression of MST3 correlates with survival of breast cancer patients **A.** Immunoblotting assay was used to assess the expression of MST3 in normal breast tissue (N) and breast tumor (T) specimens. Equal amounts (30μg) of protein from whole-tissue lystaes were analyzed for MST3 and β-actin expression by Western blotting analysis. **B.**and **C.** Quantitative analysis of the immunoblotting assay and the subtype and grade of breast cancer tissues. **D.** The MST3 level in non-triple negative and triple-negative breast cancers of the TCGA breast dataset was analyzed. **E.** Kaplan-Meier analysis for overall survival in breast cancer patients according to the expression of MST3 (*n* = 1117). Auto select best cutoff was chosen in the analysis. The best specific probes (JetSet probes) that recognized MST3 which maps Affymetrix probe sets by selecting the best probe set for this analysis. High levels of MST3 expression were associated with decreased patient survival (log-rank *P* = 0.0043), and the hazard ratio (HR) (with 95% confidence intervals) was shown. Query parameters were: overall survival, split patients by median, auto-select best cut-off and only JetSet best probe set.

To elucidate the clinical relevance of MST3 in cancer patients, we analyzed 20 breast cancer tissues by immunoblotting (Figure 1A). In 14 of 20 patients, the MST3 protein levels in breast cancer tissue were at least 1.2-fold higher than those in adjacent normal breast tissue (Figure 1B). Consistently, the expression levels of MST3 in breast cancer tissues were significantly (*p* < 0.001) higher than those in matched normal tissues by statistical analysis (Figure 1C). Figure 1B showed the molecular subtype and grade of breast cancer. Higher MST3 levels were observed in triple-negative breast cancers (TNBC) (patient 9, 15, 19, 20) than that in other molecular subtype breast cancers. We performed a meta-analysis of published gene expression data using the Oncomine database. We compared the MST3 levels of 31 TNBC cases *vs* 107 non-TNBC cases in the TCGA breast dataset. MST3 expression in TNBC cases was higher than that in non-TNBC cases (Figure 1D). We analyzed the relationship between MST3 mRNA expression and breast cancer using an online Kaplan-Meier plotter based on a public database, which contains microarray data of 22,277 genes and overall survival, relapse-free survival, and distant metastasis-free survival of 2,977 breast cancer samples [41-43]. Remarkably, MST3 expression was significantly correlated with the survival outcome of breast cancer patients. High expression of MST3 was correlated with a low survival rate in overall survival (Figure 1E) outcomes. Taken together, these data indicated that up-regulation of MST3 confers significant clinical importance and represents a predictive marker for the survival of breast cancer patients.

### Downregulation of MST3 inhibits the proliferation and tumorigenicity of triple-negative breast cancer cell lines

**Figure 2:**
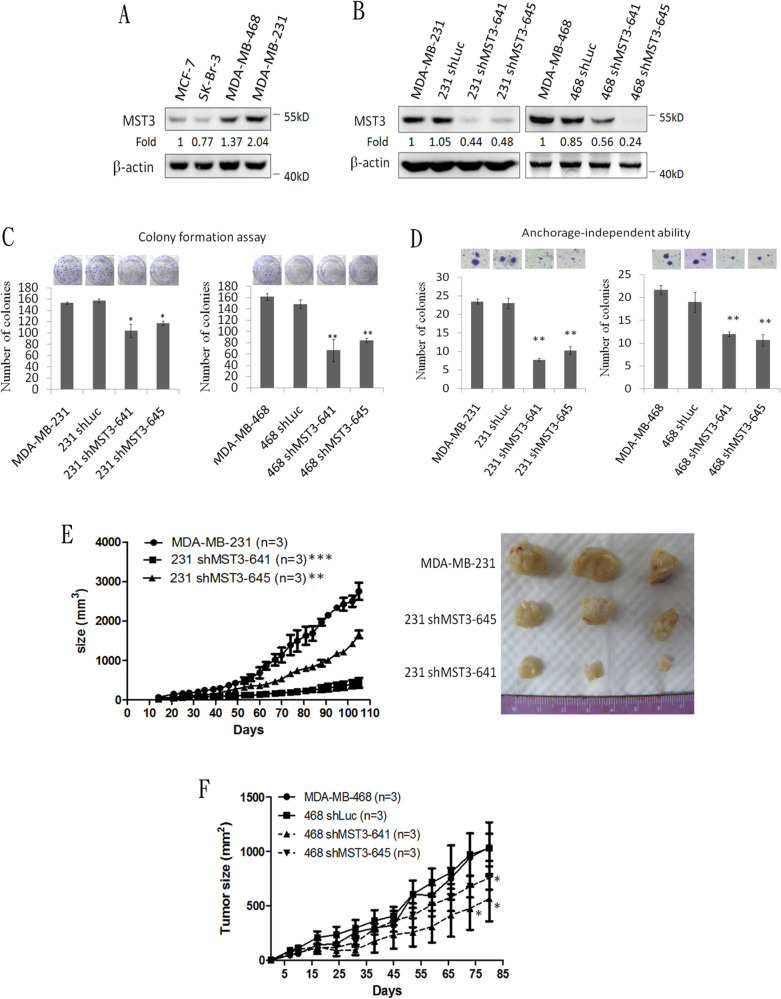
Attenuation of MST3 by shRNA inhibits proliferation, anchorage-independent growth, and tumor growth of breast cancer cells **A.** Western blotting analysis of MST3 in breast cancer cell lines. Equal amounts (30μg) of protein from whole-cell lysates were analyzed for MST3 and β-actin expression by Western blotting analysis. **B.** Western blotting analysis of MST3 in MDA-MB-231 and MDA-MB-468 cells that were stably transfected with two different MST3 shRNA plasmids (641 and 645). Equal amounts (30μg) of protein from whole-tissue lysates were analyzed for MST3 and β-actin expression by Western blotting analysis. **C.** The proliferation rates of the indicated cell lines were determined by colony formation assay. Representative images of colony formation assay were shown (upper panel). **D.** The ability of anchorage-independent growth of the indicated cell lines was determined by soft agar assay. Representative images of clone formation in soft agar were shown (upper panel). **E.** MST3 shRNA transfectants and MDA-MB-231 cells were injected s.c. into the flanks of NOD/SCID mice. After transplantation, tumor size was measured at the indicated days. Representative images of dissected tumors were shown (right panel). **F.** MST3 shRNA transfectants and MDA-MB-468 cells were injected s.c. into the flanks of NOD/SCID mice. After transplantation, tumor size was measured at the indicated days. Data are represented as mean ± SD from three independent experiments. * *p* < 0.05; **p < 0.01; *** *p* < 0.001.

To investigate whether MST3 influenced the growth of breast cancer cells, we analyzed the expression level of MST3 in four breast cancer cell lines. MST3 expression was higher in two TNBC cell lines, MDA-MB-231 and MDA-MB-468 cells than that in MCF-7 and SK-Br-3 cells, two non-TNBC cell lines (Figure 2A). Therefore, MDA-MB-231 and MDA-MB-468 cells were transfected with the plasmid containing MST3 shRNA, and stable transfectants were obtained by selection with G418. These shRNAs were designed to target the 3′UTR (TRCN0000000641) and the coding region (TRCN0000000645) of MST3. MST3 expression was reduced by MST3 shRNA in MDA-MB-231 and MDA-MB-468 cells (Figure 2B). Downregulation of MST3 expression caused a significant reduction in colony numbers in both MDA-MB-231 and MDA-MB-468 cells in the colony formation assay (Figure 2C). These results indicated that MST3 plays a significant role in the proliferation of breast cancer cells. In addition, MST3 knockdown significantly decreased the ability of anchorage-independent growth of both breast cancer cell lines (Figure 2D). To determine whether MST3 knockdown inhibited the tumorigenicity of breast cancer cells *in vivo*, MDA-MB-231 and their shMST3 stable transfectant cells were inoculated subcutaneously into the flank region of NOD/SCID mice. MDA-MB-231 shMST3 stable transfectant cells exhibited slower tumor formation than the parental cells in the NOD/SCID mice (Figure 2E). A similar result was obtained in MDA-MB-468 cells, although the effects on tumor growth were milder (Figure 2F). Therefore, our results indicate that MST3 plays an important role in promoting breast tumorigenicity both *in vitro* and *in vivo*.

### MST3 interacts with VAV2 in breast cancer cells

**Figure 3:**
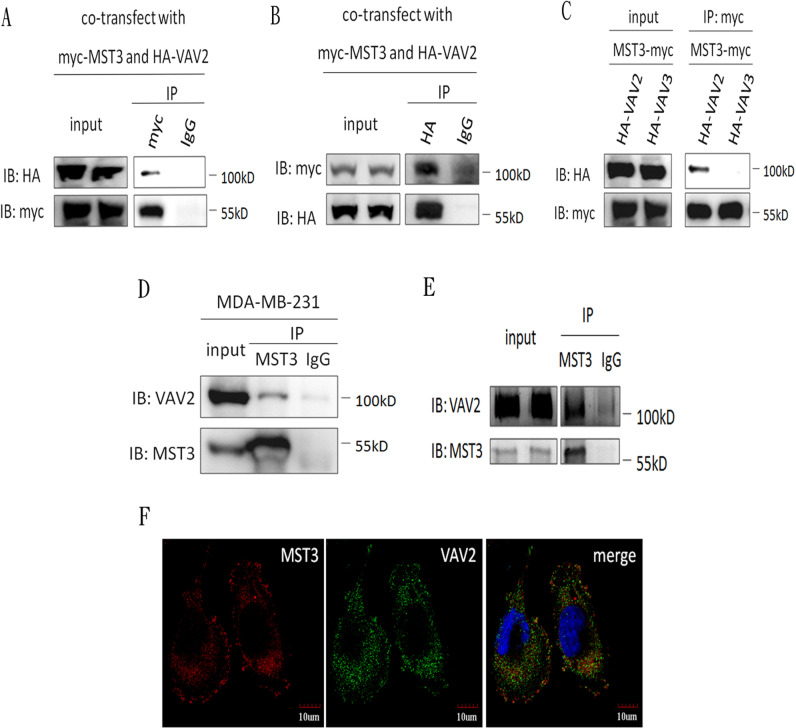
MST3 interacts with VAV2 HEK293 cells were co-transfected with MST3-myc and HA-VAV2 or HA-VAV3 **A., B.** and **C.** Equal amounts (0.5mg) of total protein were immunoprecipitated with 1μg anti-myc or anti-HA antibody. The immunoprecipitates were assessed by immunoblotting using anti-myc and anti-HA antibodies. **D.** Equal amounts (1mg) of total protein from MDA-MB-231 cells were immunoprecipitated with 1μg anti-MST3 antibody. The immunoprecipitates were assessed by immunoblotting using anti-MST3 or anti-VAV2 antibody. **E.** 0.5μg purified recombinant MST3 incubated with 1μg purified recombinant VAV2 *in vitro*. The protein mixtures were immunoprecipitated with anti-MST3 antibody. The immunoprecipitates were assessed by immunoblotting using anti-MST3 and anti-VAV2 antibody. **F.** Representative photographs for immunofluorescence staining of endogenous MST3 and VAV2 in MDA-MB-231 cells. Nuclei were stained with DAPI.

Because MST3 was suggested to be a possible interacting partner for VAV2 in a previous systematic yeast two-hybrid study, we aimed to determine whether the interaction between VAV2 and MST3 occurred *in vitro* and *in vivo*. The interaction between MST3 and VAV2 was first examined in a transfection model system. HEK293 cells were transiently transfected with HA-tagged VAV2 and myc-tagged MST3 expression plasmids, and the interaction between MST3 and VAV2 was determined by co-immunoprecipitation. Immunoprecipitation with anti-myc-MST3 pulled down HA-VAV2 (Figure 3A), and vice versa (Figure 3B). In contrast, VAV3 was not associated with myc-tagged MST3 in co-immunoprecipitation (Figure 3C). Endogenous MST3 was found to associate with VAV2 in MDA-MB-231 cells (Figure 3D). Since purified recombinant MST3 protein was co-immunprecipitated with recombinant VAV2 protein *in vitro*, direct interaction between MST3 and VAV2 was suggested (Figure 3E). Finally, the co-localization of MST3 and VAV2 in the MDA-MB-231 cells was confirmed by confocal microscopy examination (Figure 3F).

### The proline-rich region of MST3 is required for the interaction with the SH3 domains of VAV2

**Figure 4:**
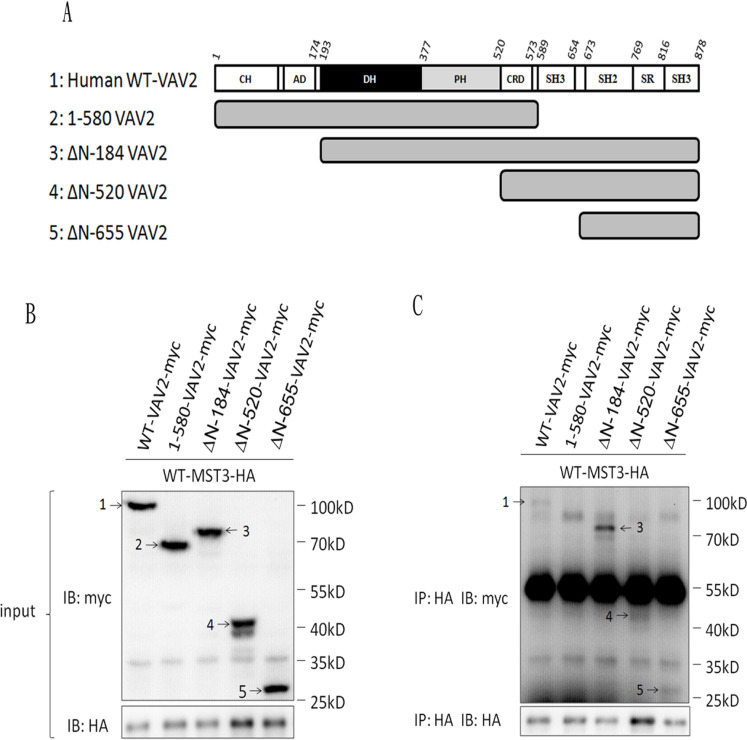
The SH3 domain of VAV2 is required for the interaction with MST3 **A.** Schematic diagram representing expressed fragments of VAV2. Wild-type VAV2-myc and fragments VAV2-myc were constructed in pcDNA3.1/myc-His vector. **B.** Myc-tagged fragments of VAV2 as indicated were co-expressed with MST3-HA in HEK293 cells. The expression of VAV2 fragments were demonstrated by western blotting. **C.** Equal amounts (0.5mg) of total protein were immunoprecipitated with 1μg anti-HA antibody. The immunoprecipitates were assessed by immunoblotting using anti-HA and anti-myc antibodies. 1: WT-VAV2-myc, 2: 1-580-VAV2-myc, 3: ΔN-184-VAV2-myc, 4: ΔN-520-VAV2-myc, 5: ΔN-655-VAV2-myc.

**Figure 5:**
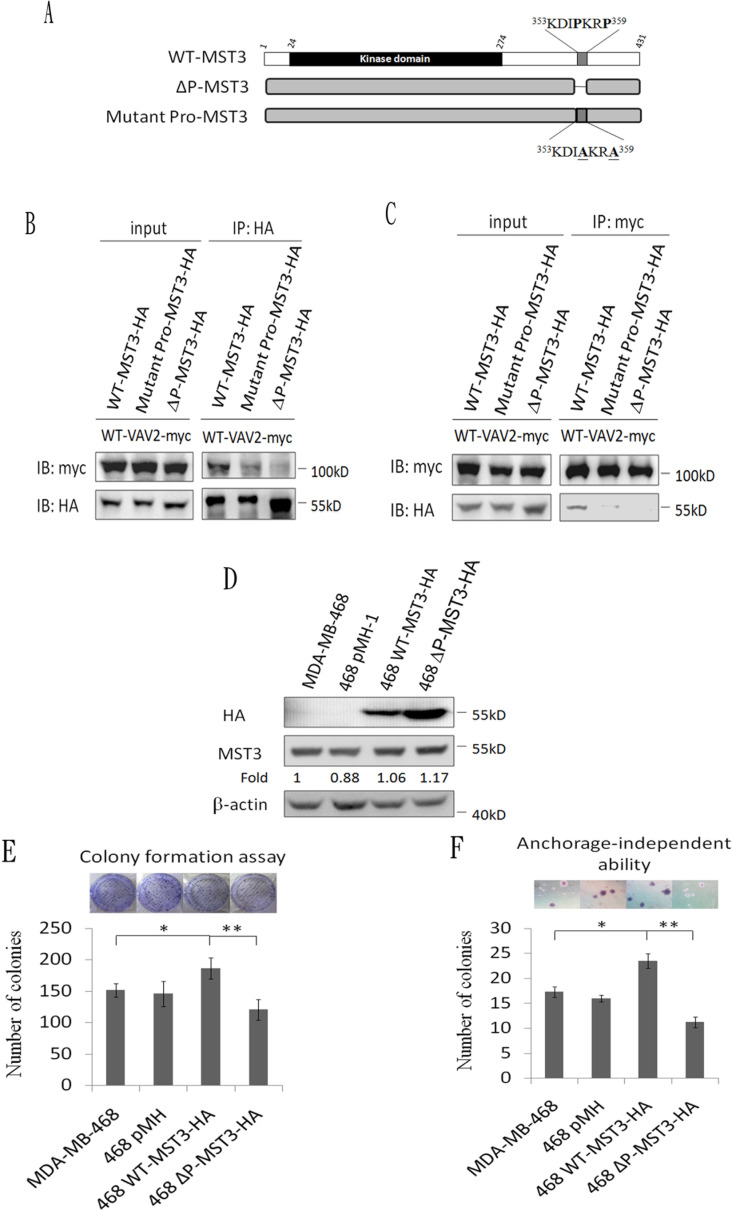
The proline-rich region of MST3 is required for binding to VAV2 and is essential for enhancing cell proliferation and growth in soft agar **A.** Schematic diagram representing wild-type and mutant MST3. **B.** and **C.** Wild-type-MST3-HA or mutant-MST3-HA (ΔP: deletion of proline-rich domain; mutant pro: proline mutation of proline-rich domain) were co-expressed with VAV2-myc in HEK293 cells. Equal amounts (0.5mg) of total protein were immunoprecipitated with 1μg anti-myc or anti-HA antibody. The immunoprecipitates were immunoblotted with anti-myc and anti-HA antibodies to determine the interaction between VAV2 and mutant MST3. **D.** Wild-type-MST3-HA or mutant MST3-HA (deletion of proline-rich domain; ΔP) was expressed in MDA-MB-468 cells. Equal amounts (30μg) of protein from whole-cell lysates were analyzed for the indicated proteins by Western blotting analysis. **E.** The proliferation rates of the indicated cell lines were determined by colony formation assay. Representative images of colony formation assay were shown (upper panel). **F.** The ability of anchorage-independent growth of the indicated cell lines was determined by soft agar assay. Representative images of clone formation in soft agar were shown (upper panel). Data are represented as mean ± SD from three independent experiments. * *p* < 0.05; ** *p* < 0.01.

Having demonstrated an interaction between MST3 and VAV2, we further mapped the interaction domain between MST3 and VAV2. Four truncated mutants of VAV2 were constructed to investigate the MST3-interaction domain (Figure 4A). MST3 could be pulled down by full-length VAV2, N-terminally deleted VAV2, and the SH2-SH3 domain of VAV2 but not by VAV2 lacking both SH3 domains (Figure 4B and 4C). It suggests that deletion of both SH3 domains renders the VAV2 unable to interact with MST3. It is interesting to note that deletion of N-terminal domain confers stronger ability to interact with MST3, suggesting that a potential inhibitory function resides on the N-terminal region of VAV2 (Figure 4C). The SH3 domain generally binds to a proline-rich region. Two recognized motifs of a proline-rich region are frequently observed: [RKHYFW]xxPxxP (class I) and PxxPx[RK] (class II) [44]. Based on this information, we hypothesized that the proline-rich region (^353^KDIPKRP^359^) of MST3 might be essential for interacting with VAV2, and we constructed two plasmids expressing MST3 with deletion of the proline-rich domain (ΔP-MST3) or mutations on two proline residues in the proline-rich domain (mutant Pro-MST3) (Figure 5A). In the co-immunoprecipitation experiment, mutant Pro-MST3 interacted very weakly with VAV2, and ΔP-MST3 failed to bind VAV2. (Figure 5B and 5C). Therefore, the two proline residues in proline-rich region of MST3 is essential for the interaction with VAV2. Overexpression of WT-MST3, but not ΔP-MST3, significantly increased colony numbers with colony formation assay (Figure 5E) and soft agar assay (Figure 5F) in TNBC MDA-MB-468 cells. These data indicate that the proline-rich region of MST3 is required for cell proliferation and anchorage-independent growth, and suggest the interaction between MST3 and VAV2 is important for oncogenic function of MST3.

### The interaction of MST3 with VAV2 enhances cell growth and activation of the VAV2-Rac1 pathway

**Figure 6:**
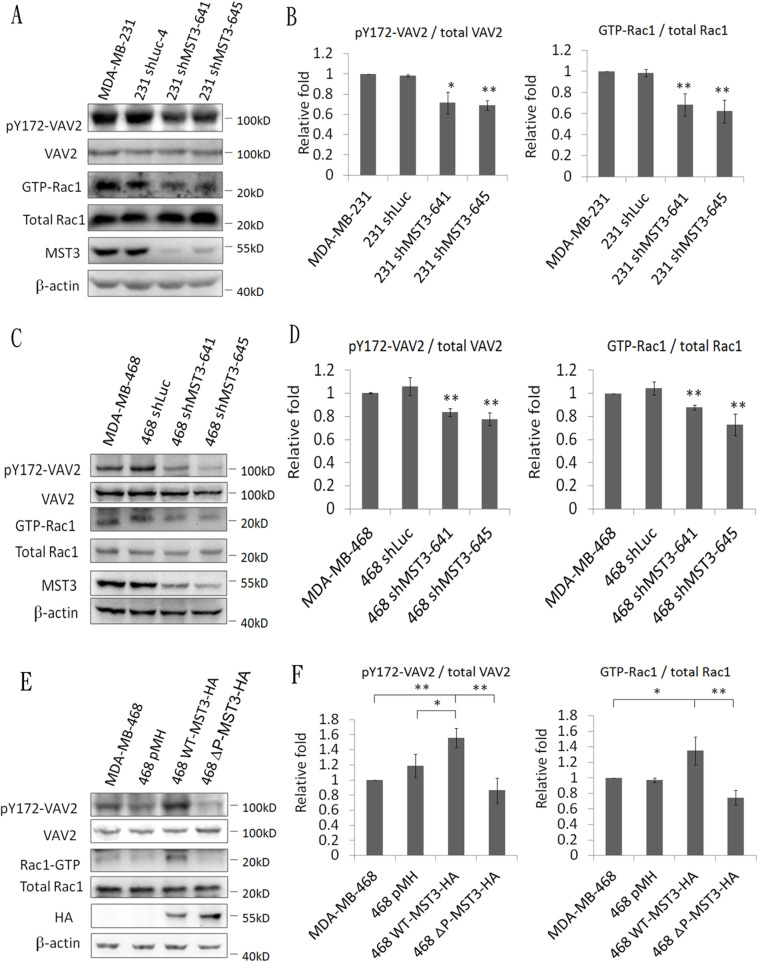
MST3 enhances VAV2 phosphorylation and Rac1 activation **A.** and **C.** MST3 shRNA was stably expressed in MDA-MB-231 and MDA-MB-468 cells. **E.** Wild-type-MST3-HA or mutant MST3-HA (deletion of proline rich domain; ΔP) was expressed in MDA-MB-468 cells. Equal amounts (30μg) of protein from whole-cell lysates were analyzed for VAV2, ectopic MST3 protein (HA), endogenous MST3, and β-actin expression by Western blotting analysis. 50μg total proteins from whole-cell lysates were analyzed by immunoblotting with antibody specific to phosphorylated VAV2. GTP-Rac1 was assayed using a Rac1 Activation Assay kit. **B.**, **D.** and **F.** Quantification and statistical analysis of pVAV2 and GTP-Rac1. Data are represented as mean ± SD from three independent experiments. *p < 0.05; ** *p* < 0.01.

The activation of VAV2 involves phosphorylation on tyrosine residues, leading to the activation of their GDP/GTP exchange activity toward Rho/Rac1 protein [20, 45]. Hence, we examined whether the association between MST3 and VAV2 affected VAV2 phosphorylation. Decreased phosphorylation on Y172 of VAV2 was evident in MDA-MB-231 and MDA-MB-468-shMST3 stable transfectant cells. In addition, knockdown of MST3 attenuated GTP-Rac1 expression (Figure 6A to 6D, supplementary 1A and 1B). MDA-MB-468 cells have a lower endogenous MST3 expression than that in with MDA-MB-231 cells. Hence, we expressed ectopic WT- or ΔP-MST3 in low-MST3-expression MDA-MB-468 cells (Figure 2A). Enhanced phosphorylation of VAV2 and Rac1 activation were observed in WT-MST3 stable transfectants but not in ΔP-MST3 stable transfectants (Figure 6E, 6F and supplementary 1C). These data suggest that the proline-rich region in MST3 was required to enhance the activation of the VAV2-Rac1 pathway, further supporting that interaction between the proline-rich domain and SH3 domain has a functional consequence.

### MST3 induces cyclin D1 expression through the VAV2-Rac1 pathway to promote cell growth and tumorigenesis

**Figure 7:**
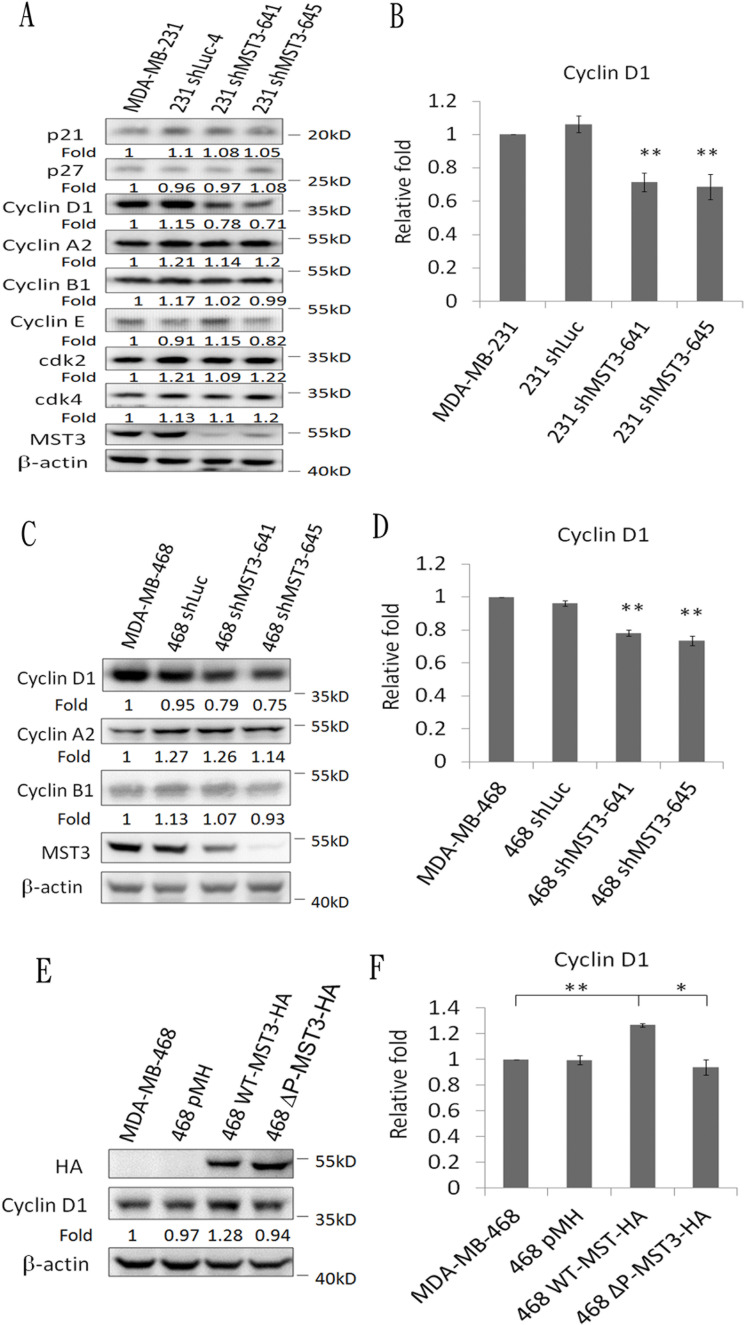
Downregulation and overexpression of MST3 regulate cyclin D1 expression Immunoblotting analysis of the cell-cycle-related molecules and MST3 in **A.** MDA-MB-231-shMST3 **C.** MDA-MB-468-shMST3 and **E.** MST3-overexpression stable transfectants. Equal amounts (30μg) of protein from whole-cell lysates were analyzed for the indicated proteins by Western blotting analysis. **B.**, **D.** and **F.** Quantification and statistical analysis of cyclin D1. Data are represented as mean ± SD from three independent experiments. * *p* < 0.05, ** *p* < 0.01.

**Figure 8:**
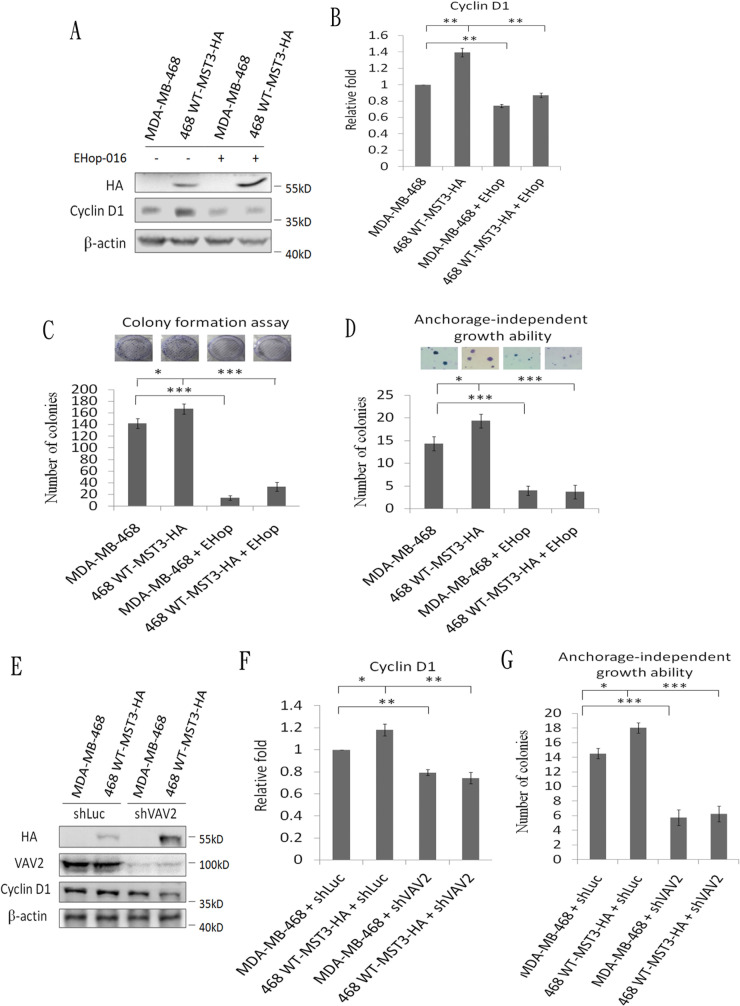
MST3 induces cyclin D1 through the VAV2-Rac1 pathway to promote cell growth and tumorigenesis **A.** MST3-overexpression stable transfectants were treated with 2 μM of the Rac1 inhibitor EHop-016. Equal amounts (30μg) of protein from cell lysates were analyzed for cyclin D1, HA, and β-actin expression by Western blotting analysis. **C.** The proliferation rate of the indicated cell lines were determined by colony formation assay. **D.** The ability of anchorage-independent growth of the indicated cell lines was determined by soft agar assay. Representative images of clone formation in soft agar were shown (upper panel). **E.** MST3-overexpression stable transfectants were infected with shLuc and shVAV2 lentivirus. Equal amounts (30μg) of protein from whole-cell lysates were analyzed for cyclin D1, HA, VAV2, and β-actin expression by Western blotting analysis. **G.** The ability of anchorage-independent growth of the indicated cell lines was determined by soft agar assay. **B.** and **F.** Quantification and statistical analysis of cyclin D1. Data are represented as mean ± SD from three independent experiments. *p < 0.05, ** *p* < 0.01, *** *p* < 0.001.

**Figure 9:**
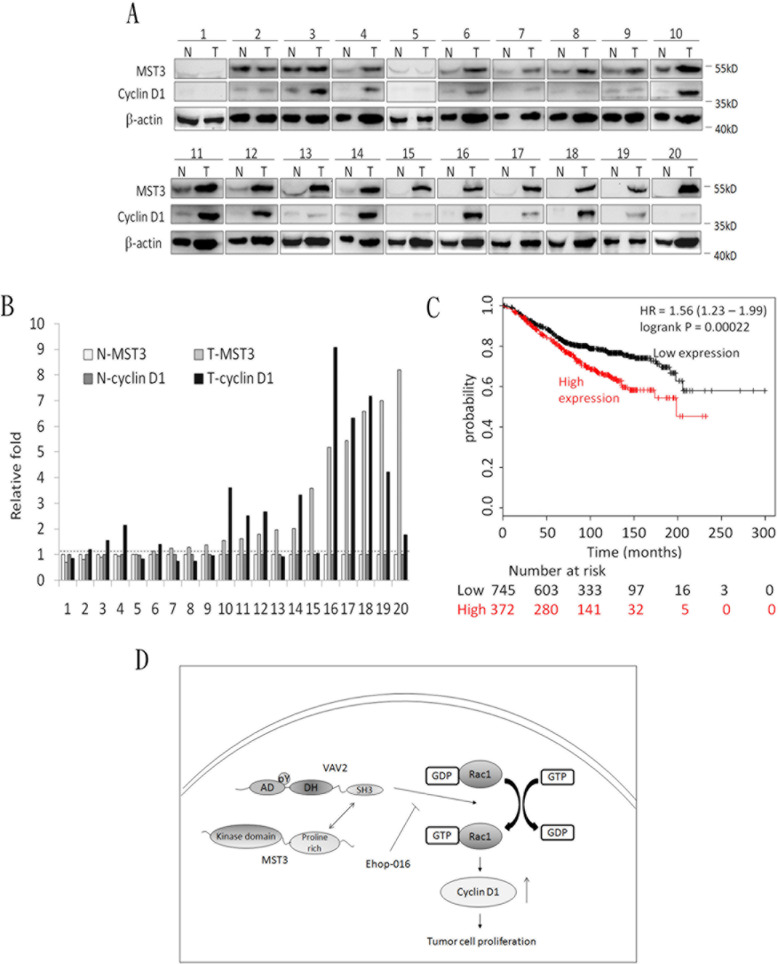
The co-overexpression of MST3 and cyclin D1 in breast tumor tissues **A.** Immunoblotting assay was used to determine the expression of MST3 and cyclin D1 in normal breast tissue (N) and breast tumor (T) specimens. Equal amounts (30μg) of protein from whole-tissue lysates were analyzed for MST3, cyclin D1 and β-actin expression by Western blotting analysis. **B.** Quantitative analysis of the MST3 and cyclin D1 protein level in normal breast tissue (N-MST3 and N-cyclin D1) and breast tumor (T-MST3 and T-cyclin D1) specimens. **C.** Kaplan-Meier analysis for overall survival in breast cancer patients (*n* = 1117) according to the co-expression of MST3 and cyclin D1. Auto select best cutoff was chosen in the analysis. The best specific probes (JetSet probes) that recognized MST3 and cyclin D1 which maps Affymetrix probe sets by selecting the best probe set for this analysis. High-level co-expression of MST3 and cyclin D1 was associated with decreased patient survival (log-rank *P* = 0.00022), and the hazard ratio (HR) (with 95% confidence intervals) was shown. **D.** Model for MST3 function in the VAV2/Rac1 signaling axis. The interaction of MST3 and VAV2 enhances tyrosine phosphorylation and activity of VAV2. Increased VAV2 activity activates Rac1-GTPase and induces cyclin D1 expression to promote tumor growth.

A previous report has suggested that Rac1 contributes to cancer cell proliferation *via* cyclin D1 induction [46]. We observed that cyclin D1 was significantly reduced in MDA-MB-231 and MDA-MB-468 shMST3 stable transfectants (Figure 7A to 7D). Moreover, cyclin D1 was further enhanced by the overexpression of WT-MST3 but not by that of ΔP-MST3 (Figure 7E and 7F). These data indicate that the proline-rich region of MST3 is required for cyclin D1 induction. We then investigated whether MST3 induced cyclin D1 expression through the VA2-Rac1 pathway. The Rac1 inhibitor EHop-016 blocked the interaction of VAV2 with Rac1 and inhibited Rac1 activation at low concentrations [47]. EHop-016 attenuated cyclin D1 expression that was induced by MST3 (Figure 8A and 8B). In addition, EHop-016 decreased the anchorage-dependent growth and anchorage-independent growth with a colony formation assay (Figure 8C) and a soft agar assay (Figure 8D) in MDA-MB-468 cells. Downregulation of VAV2 by shRNA reduced cyclin D1 expression and the anchorage-independent growth ability of WT-MST3 stable transfectants (Figure 8E and 8G). Thus, MST3 induced cyclin D1 expression and cell growth mainly through the VA2-Rac1 pathway. Finally, cyclin D1 was co-overexpressed in nine of 14 breast cancer tissues with MST3 overexpression (Figure 9A and 9B). High-level coexpression of MST3 and cyclin D1 was observed in human breast cancer and was correlated with poor overall survival by using the Kaplan-Meier plotter (Figure 9C).

## DISCUSSION

In this report, we demonstrated that MST3 plays an oncogenic role in breast cancer, especially in triple-negative breast cancers. Online Kaplan-Meier plotter analysis revealed that overexpression of MST3 predicts a poor prognosis in breast cancer patients. Decreased cell growth and anchorage-independent growth are observed in the MST3 shRNA stable transfectants. MST3 was shown to interact with VAV2, and the proline-rich domain of MST3 was suggested to interact with the SH3 domains of VAV2. Overexpression of wild-type MST3 enhances the proliferation rates and anchorage-independent growth through the VAV2-Rac1-cyclin D1 pathway. In contrast, mutant MST3 devoid of proline-rich domain lost the ability to promote cell growth and anchorage-independent growth. These observations indicated a novel signaling pathway in which MST3 interacts with VAV2 to induce cyclin D1 and promote the tumorigenicity of breast cancer (Figure 9D). Furthermore, downregulation of MST3 in triple-negative MDA-MB-231 and MDA-MB-468 breast cancer cells delayed tumor growth in NOD/SCID mice. These results also provide new insights into the potential therapeutics in triple-negative breast cancer, which lacks the expression of the estrogen receptor, progesterone receptor, and HER2.

In our study, MST3 is over-expressed in clinical breast cancer tissues and MST3 expression is correlated with the survival outcome of breast cancer patients. In addition, attenuation of MST3 by shRNA inhibits the tumorigenesis of breast cancer cells *in vitro* and *in vivo*. Herein, we first demonstrated that MST3 plays a potential oncogenic role in human breast cancer. Previous study by other groups and our group have indicated that the truncated MST3 is translocated into nucleus to induce apoptosis [4,5]. In addition, MST3 plays a role in apoptosis of trophoblast cell lines [7]. However, MST3 was shown to induce growth through NDR protein kinase [9]. It is possible that the location and complex partners may be very important factors in the biological function of MST3. In our study, MST3 interacts with oncoprotein VAV2 in the cytoplasm and cell membrane, as demonstrated by confocal microscopy. The role of MST3 in human cancers maybe cell-type dependent and cell-context dependent.

MST3 expression levels in 20 breast cancer tissues were mostly (14 pairs) higher than those in adjacent normal breast tissue. However, breast cancer may progress through multiple pathways. Other oncogenic molecules, including HER2, Ras, and Myc, may be mainly responsible for the tumorigenicity in other six breast tissues. Besides, MST3 was overexpressed in 14 breast cancer tissues, but MST3 expression level does not correlate with the stages of breast cancer. It is possible that overexpression of MST3 is required in early oncogenesis of breast cancer, and that the oncogenic role of MST3 is compensated by other gene products in later stages of breast cancer. Therefore, the expression level of MST3 may be lower in the advanced stage of breast cancer.

A previous study reported that the MST3-NDR signaling pathway promotes cell cycle progression by reducing p21 stability in HeLa cells [48]. In our study, downregulation of MST3 did not increase the protein level of p21 in MDA-MB-231 cells (Figure 7A). Thus, MST3 may form different complexes in various cancer cells to promote tumorigenesis. We first identified the proline-rich domain of MST3 as the essential interaction domain for VAV2. There are two recognized motifs of the proline-rich region: class I- [RKHYFW]xxPxxP and class II-PxxPx[RK]. The proline-rich domain of MST3 (^353^KDIPKRP^359^) belongs to the class I proline-rich motif. Interestingly, another GCK family kinase HPK1 binds to the SH3 domains of Crk, CRKL and Grb2 adaptor proteins through a class II proline-rich domain [49].

Our results indicate that the interaction of MST3 and VAV2 enhances VAV2 activation. Similarly, a previous study showed that Nek3, a serine/threonine kinase, contributes to prolactin receptor-mediated VAV2 serine phosphorylation and facilitates VAV2 tyrosine phosphorylation [50]. MST3 is a serine/threonine kinase, unable to act directly on the tyrosine residue of VAV2. Tyrosine phosphorylation of VAV protein is required for its activation by receptor protein tyrosine kinase or tyrosine kinase Src. MST3 might be involved in an RTK-Src-VAV2 complex to enhance VAV2 activation; however, further experiments are required for analyzing a total complex formation.

The overexpression of cyclin D1 has been linked to the development and progression of breast cancer. In this report, cyclin D1 was co-overexpressed in nine of 14 breast cancer tissues with MST3 overexpression, but cyclin D1 expression level does not correlate to that of MST3 expression level. Previous studies indicated that GSK3β and the Skp-Cullin-F-box (SCF) E3 ubiquitin ligase complexes catalyze cyclin D1 degradation [51-52]. Kinase-inactive GSK3β in murine mammary glands can promote breast cancer development [53]. Cyclin D1 induction through MST3-VAV2-Rac1 cascade may be further degraded by GSK3β or the SCF complexes. Our results suggest that MST3/VAV2/cyclin D1 is one of the important oncogenic pathways in breast cancer development, but breast cancer development requires a coordination of MST3 pathway and other oncogenic pathways.

In summary, MST3 plays a potential oncogenic role in breast cancer. Upregulation of MST3 has a significant clinical correlation and represents a predictive marker for the survival of breast cancer patients. MST3 induces cyclin D1 by binding VAV2 and enhancing Rac1 activation to promote the tumorigenicity of breast cancer. Downregulation of MST3 inhibits proliferation and tumorigenicity in triple-negative breast cancer cell lines, suggesting a new potential therapeutic option.

## MATERIALS AND METHODS

### Antibodies

Anti-HA (#11867431001) antibody was purchased from Roche Applied Science (Mannheim, Germany). Anti-myc (OP10) and anti-Rac1 (#05-389) antibodies were purchased from Merck Millipore (Darmstadt, Germany). Anti-VAV2 (#1993-1), anti-cyclin E (#1655) and anti-VAV2-PY172 (ab8669) antibody were purchased from Abcam (Cambridge, Massachusetts, USA). Anti-p21 antibody was purchased from Genetex (Irvine, California, USA). Anti-p27 (#2552), anti-cyclin D1 (#2926), anti-cdk2 (#2545) and anti-cdk4 (#2906) antibodies were purchased from Cell Signaling (Danvers, Massachusetts, USA). Anti-cyclin A2 (SC-596) antibody was purchased from Santa Cruz Biotechnology (Santa Cruz, California, USA). Anti-β-actin (MAB1501R) antibody was purchased from Chemicon International (Temecula, CA, USA). Anti-MST3 (#611057) monoclonal antibody was purchased from BD Transduction Laboratories (Franklin Lakes, New Jersey, USA). Anti-MST3 polyclonal antibody was prepared as described previously.

### Plasmids and shRNA-lentivirus

MST3, VAV2 and luciferase shRNA in pLKO.1 were purchased from the National RNAi Core Facility (Academia Sinica, Taiwan). The catalog numbers and shRNA sequences were TRCN0000000641(5′-CCGGGCAGGGTTTGTCATTAATAATCTCGAGATTATTAATGACAAACCCTGCTTTTT-3′) for the 3′UTR region of human MST3 mRNA; TRCN0000000645(5′-CCGGTGCAGAGTTGAAGGAGAAGAGCTCGAGCTCTTCTCCTTCAACTCTGCATTTTT-3′) for the coding region of human MST3 mRNA; TRCN0000048227(5′-CCGGCAAGTGAAACTGGAGGAATTTCTCGAGAAATTCCTCCAGTTTCACTTGTTTTTG-3′) for human VAV2 mRNA; TRCN0000231741(5′-CCGGGCTGAGTACTTCGAAATGTCCCTCGAGGGACATTTCGAAGTACTCAGCTTTTTG-3′) for luciferase. pC.HA-VAV2, and pC.HA-VAV3 plasmids were purchased from Addgene. Wild-type VAV2-myc and the fragment VAV2-myc were constructed between the Hind III and EcoR I sites in pcDNA3.1/myc-His (Invitrogen Life Technologies). Wild-type MST3-myc was constructed between the Hind III and EcoR I sites in pcDNA3.1/myc-His. Wild-type MST3-HA was constructed between the Hind III and EcoR I sites in pMH (Roche Applied Science). The deletion of the proline-rich domain of MST3-HA was performed by inserting 1-344 and 361-431 MST3 fragments between the Hind III, EcoR I and Not I sites in pMH. The plasmid expressing MST3 with proline mutations in the proline-rich domain (mutant Pro-MST3) was achieved by PCR-directed mutagenesis. The primers for mutant Pro-MST3 were as follows: forward primer, 5′-GATGAAAGACATCGCAAAGAGGGCTTTCTCTCAGTGTTTATC-3′; reverse primer, 5′-GATAAACACTGAGAGAAAGCCCTCTTTGCGATGTCTTTCATC-3′. The underlined base pairs represent the mutated amino acids. VAV2 and luciferase shRNA-lentivirus were purchased from National RNAi Core Facility.

### Kaplan-Meier curves

The correlation between the expression of genes and prognosis of breast cancer patients was analyzed using an online Kaplan-Meier plotter (http://kmplot.com/analysis/). The Kaplan-Meier plotter is a competent tool for assessing the effect of any gene or gene combination on survival in breast, lung, ovarian and gastric cancer patients using 10,188 cancer samples. The datasets include gene expression and survival data from Gene Expression Omnibus (GEO) and The Cancer Genome Atlas (TCGA) (Affymetrix HG-U133A, HG-U133A 2.0, and HG-U133 Plus 2.0 microarrays). Our results were collected from 1117 breast cancer patients. To analyze the prognostic value of the probe, the samples were split into two groups according to the median expression of the probe (auto select best cutoff). The two patient groups (higher and lower expression of genes) were compared using a Kaplan-Meier survival plot. The hazard ratios (HRs) with 95% confidence intervals, and the log rank p value was calculated using a default algorithm as previously described [41]. We analyzed the best specific probes (JetSet probes) that recognized genes which maps to Affymetrix probe sets by selecting the best probe set for this analysis [54].

### Oncomine gene expression data analysis

Relative levels of *MST3* mRNA expression in human breast cancer were investigated by Oncomine Cancer Microarray database analysis (www.oncomine.org) [55] of The Cancer Genome Atlas (TCGA) database. Oncomine algorithms were used for the statistical analysis of the differences in *MST3* mRNA expression.

### Sample tissues

Tissue specimens from 20 patients with breast cancer were diagnosed histopathologically and clinically at National Cheng Kung University Hospital (Tainan, Taiwan). Pathological stage of breast cancer was performed in accordance with the 7th edition of the American Joint Committee on Cancer (AJCC) staging system [56]. Prior patient consent and approval from the Institutional Review Board were obtained for the use of these clinical tissues for research purposes (NCKUH IRB number: ER-98-092). These sample tissues were collected during excision surgery.

### Cell culture

Human breast adenocarcinoma MDA-MB-231 cells and HEK293 cells were cultured in defined minimal essential medium (DMEM) supplemented with 10% fetal bovine serum (FBS), 100 μg/ml streptomycin, and 100 U/ml penicillin at 37°C and 5% CO_2_. Human breast adenocarcinoma MDA-MB-468 cells were maintained in RPMI 1640 containing 10% FBS and antibiotics at 37°C and 5% CO_2_.

### Protein extraction

Cultured cells and sample tissues were homogenized in Triton X-100 lysis buffer (pH 7.8, containing 50 mM Tri-HCl, 150 mM NaCl, 1% Triton X-100 and protease inhibitors). The lysates centrifuged at 16,000 × g for 10 min at 4°C. The supernatant was collected after centrifugation, and the protein concentrations were determined using a Micro BCA^TM^ protein assay kit (Pierce).

### Western blotting analysis

Equal amounts (30-50μg) of protein were electrophoretically separated in the SDS polyacrylamide gel electrophoresis (PAGE) and transferred to a polyvinylidene difluoride (PVDF) membrane, using a Hofer transfer cassette, for 90 min. The membranes were then incubated with a blocking buffer of 5% nonfat dried milk dissolved in TBST buffer for 1 hr at room temperature, and then incubated with the indicated antibodies overnight at 4°C. The membranes were washed three times with TBST buffer and then incubated with appropriate secondary antibodies for 1 hr followed by washing three times. The proteins on the blots were detected using ECL Western blotting detection reagents and captured by a BioSpectrum AC imaging system (UVP).

### Co-immunoprecipitation

Cells were lysed in Triton X-100 lysis buffer. Equal amounts (0.5-1mg) of total protein were precleared by incubation with 20 μl of protein G-Sepharose for 1 hr at 4°C. The precleared supernatants were subjected to overnight immunoprecipitation using the indicated antibodies or control IgG antibodies at 4°C, followed by the addition of 40 μl of protein G-Sepharose for 1 hr at 4°C. The immunoprecipitates were washed three times with Triton X-100 lysis buffer, denatured with 20μl 4× SDS loading buffer, and separated by SDS-PAGE and transblotted onto a PVDF membrane.

### *In vitro* pull-down assay

For *in vitro* pull-down assays, 0.5 μg of baculovirus-expressed MST3 [57] and 1μg of the recombinant VAV2 protein (OriGene, Rockville MD, USA) were mixed in 100 μl of phosphate-buffered saline (PBS) containing protease inhibitors, and incubated at room temperature for 10 min. Protein mixture was added to 0.5μg of anti-MST3 monoclonal antibody or control IgG antibodies, and incubated at 4°C for 3hr, followed by the addition of 40μl of protein G-Sepharose for 1 hr at 4°C. The immunoprecipitates were washed three times with Triton X-100 lysis buffer, denatured with 20μl 4X SDS loading buffer, and separated by SDS-PAGE and transblotted onto a PVDF membrane.

### Colony formation assays

Cells were seeded in 6-well plates (200 cells per well) and cultured for ten days. The colonies were fixed with 3.7% paraformaldehyde for 10 min and then stained with 0.05% crystal violet for 10 min. The number of colonies was counted for statistical analysis.

### Anchorage-independent growth assay

Five thousand cells were suspended in 1 ml of complete medium plus 0.3% agarose. The agar-cell mixture was plated onto 1.5 ml of 0.6% agarose in a 6-well plate. After 14 days, viable colonies were stained with 0.05% crystal violet and photographed in a ten random fields and scored for statistical analysis.

### Xenograft tumor model

Female NOD/SCID mice (6-7 weeks of age) were purchased from the Laboratory Animal Center at National Cheng Kung University, and housed in barrier facilities on a 12-hr light/dark cycle. All experimental procedures were approved by the Animal Welfare Committee of National Cheng Kung University. The NOD/SCID mice were injected subcutaneously into the flank region with MDA-MB-231 (5×10^5^ cells), MDA-MB-468 (5×10^6^ cells) and their shMST3 stable transfectant cells in 0.25 ml of phosphate-buffered saline (PBS). Tumor sizes were measured with calipers twice weekly; the tumor volume was calculated using the following formula: tumor volume (millimeters cubed) = L × W^2^ /2, where L is the length, and W is the width.

### Confocal immunofluorescence microscopy

Cells were grown on cover glass and fixed in 3.7% paraformaldehyde for 10 min, after which they were permeabilized with 0.1% Triton X-100 in PBS. The permeabilized cells were washed three times with PBS and then stained with primary antibody followed by secondary Alexa 488- or 594-conjugated mouse or rabbit antibodies. The nuclei were stained with DAPI. Images were photographed using fluorescence or confocal microscopy (Olympus FluoView FV1000). The images were taken in a sequential scanning mode.

### Rac1 GTPase activity pulldown assay

Assays were performed using a Rac1 Activation Assay kit, according to the manufacturer's recommendations (Millipore). Cells were cultured to approximately 70% confluence and then serum-starved for 24 hr. Rac1 activation was provoked by adding complete culture medium for 24 hr. The cells were lysed in Mg^2+^ lysis buffer containing protease inhibitors and phosphatase inhibitors, followed by centrifugation to remove cell debris. Equal amounts (0.5mg) of total protein were incubated with 10 μl of agarose-conjugated p21-binding domain of PAK1, which binds activated Rac1, for 1 hr at 4°C. The agarose beads were washed three times in lysis buffer, resuspended in 20μl 4× SDS loading buffer, and boiled for 10 min. Active (GTP-bound) and total Rac1 were analyzed by Western blotting.

### Statistical analysis

All statistical analyses were carried out using GraphPad Prism version 4.00. Student's *t* -test was used for the analysis of the differences between experimental groups.

## SUPPLEMENTARY MATERIAL FIGURE



## References

[1] Dan I, Watanabe NM, Kusumi A (2001). The Ste20 group kinases as regulators of MAP kinase cascades. Trends Cell Biol.

[2] Ramer SW, Davis RW (1993). A dominant truncation allele identifies a gene, STE20, that encodes a putative protein kinase necessary for mating in Saccharomyces cerevisiae. Proc Natl Acad Sci U S A.

[3] Ling P, Lu TJ, Yuan CJ, Lai MD (2008). Biosignaling of mammalian Ste20-related kinases. Cell Signal.

[4] Huang CY, Wu YM, Hsu CY, Lee WS, Lai MD, Lu TJ, Huang CL, Leu TH, Shih HM, Fang HI, Robinson DR, Kung HJ, Yuan CJ (2002). Caspase activation of mammalian sterile 20-like kinase 3 (Mst3). Nuclear translocation and induction of apoptosis. J Biol Chem.

[5] Lee WS, Hsu CY, Wang PL, Huang CY, Chang CH, Yuan CJ (2004). Identification and characterization of the nuclear import and export signals of the mammalian Ste20-like protein kinase 3. FEBS Lett.

[6] Martin DD, Vilas GL, Prescher JA, Rajaiah G, Falck JR, Bertozzi CR, Berthiaume LG (2008). Rapid detection, discovery, and identification of post-translationally myristoylated proteins during apoptosis using a bio-orthogonal azidomyristate analog. FASEB J.

[7] Lin CY, Wu HY, Wang PL, Yuan CJ (2010). Mammalian Ste20-like protein kinase 3 induces a caspase-independent apoptotic pathway. Int J Biochem Cell Biol.

[8] Stegert MR, Hergovich A, Tamaskovic R, Bichsel SJ, Hemmings BA (2005). Regulation of NDR protein kinase by hydrophobic motif phosphorylation mediated by the mammalian Ste20-like kinase MST3. Mol Cell Biol.

[9] Cornils H, Kohler RS, Hergovich A, Hemmings BA (2011). Human NDR kinases control G(1)/S cell cycle transition by directly regulating p21 stability. Mol Cell Biol.

[10] Lu TJ, Lai WY, Huang CY, Hsieh WJ, Yu JS, Hsieh YJ, Chang WT, Leu TH, Chang WC, Chuang WJ, Tang MJ, Chen TY, Lu TL, Lai MD (2006). Inhibition of cell migration by autophosphorylated mammalian sterile 20-like kinase 3 (MST3) involves paxillin and protein-tyrosine phosphatase-PEST. J Biol Chem.

[11] Ultanir SK, Yadav S, Hertz NT, Oses-Prieto JA, Claxton S, Burlingame AL, Shokat KM, Jan LY, Jan YN (2014). MST3 Kinase Phosphorylates TAO1/2 to Enable Myosin Va Function in Promoting Spine Synapse Development. Neuron.

[12] Glantschnig H, Rodan GA, Reszka AA (2002). Mapping of MST1 kinase sites of phosphorylation. Activation and autophosphorylation. J Biol Chem.

[13] Deng Y, Pang A, Wang JH (2003). Regulation of mammalian STE20-like kinase 2 (MST2) by protein phosphorylation/dephosphorylation and proteolysis. J Biol Chem.

[14] Fuller SJ, McGuffin LJ, Marshall AK, Giraldo A, Pikkarainen S, Clerk A, Sugden PH (2012). A novel non-canonical mechanism of regulation of MST3 (mammalian Sterile20-related kinase 3). Biochem J.

[15] Tang J, Ip JP, Ye T, Ng YP, Yung WH, Wu Z, Fang W, Fu AK, Ip NY (2014). Cdk5-dependent Mst3 phosphorylation and activity regulate neuronal migration through RhoA inhibition. J Neurosci.

[16] Sung V, Luo W, Qian D, Lee I, Jallal B, Gishizky M (2003). The Ste20 kinase MST4 plays a role in prostate cancer progression. Cancer Res.

[17] Xiong W, Knox AJ, Xu M, Kiseljak-Vassiliades K, Colgan SP, Brodsky KS, Kleinschmidt-Demasters BK, Lillehei KO, Wierman ME (2015). Mammalian Ste20-like kinase 4 promotes pituitary cell proliferation and survival under hypoxia. Mol Endocrinol.

[18] Sun T, Aceto N, Meerbrey KL, Kessler JD, Zhou C, Migliaccio I, Nguyen DX, Pavlova NN, Botero M, Huang J, Bernardi RJ, Schmitt E, Hu G, Li MZ, Dephoure N, Gygi SP (2011). Activation of multiple proto-oncogenic tyrosine kinases in breast cancer *via* loss of the PTPN12 phosphatase. Cell.

[19] Madsen CD, Hooper S, Tozluoglu M, Bruckbauer A, Fletcher G, Erler JT, Bates PA, Thompson B, Sahai E (2015). STRIPAK components determine mode of cancer cell migration and metastasis. Nat Cell Biol.

[20] Abe K, Rossman KL, Liu B, Ritola KD, Chiang D, Campbell SL, Burridge K, Der CJ (2000). Vav2 is an activator of Cdc42, Rac1, and RhoA. J Biol Chem.

[21] Movilla N, Bustelo XR (1999). Biological and regulatory properties of Vav-3, a new member of the Vav family of oncoproteins. Mol Cell Biol.

[22] Schuebel KE, Movilla N, Rosa JL, Bustelo XR (1998). Phosphorylation-dependent and constitutive activation of Rho proteins by wild-type and oncogenic Vav-2. EMBO J.

[23] Fujikawa K, Inoue Y, Sakai M, Koyama Y, Nishi S, Funada R, Alt FW, Swat W (2002). Vav3 is regulated during the cell cycle and effects cell division. Proc Natl Acad Sci U S A.

[24] Schuebel KE, Bustelo XR, Nielsen DA, Song BJ, Barbacid M, Goldman D, Lee IJ (1996). Isolation and characterization of murine vav2, a member of the vav family of proto-oncogenes. Oncogene.

[25] Zeng L, Sachdev P, Yan L, Chan JL, Trenkle T, McClelland M, Welsh J, Wang LH (2000). Vav3 mediates receptor protein tyrosine kinase signaling, regulates GTPase activity, modulates cell morphology, and induces cell transformation. Mol Cell Biol.

[26] Fernandez-Zapico ME, Gonzalez-Paz NC, Weiss E, Savoy DN, Molina JR, Fonseca R, Smyrk TC, Chari ST, Urrutia R, Billadeau DD (2005). Ectopic expression of VAV1 reveals an unexpected role in pancreatic cancer tumorigenesis. Cancer Cell.

[27] Hornstein I, Pikarsky E, Groysman M, Amir G, Peylan-Ramu N, Katzav S (2003). The haematopoietic specific signal transducer Vav1 is expressed in a subset of human neuroblastomas. J Pathol.

[28] Bartolome RA, Molina-Ortiz I, Samaniego R, Sanchez-Mateos P, Bustelo XR, Teixido J (2006). Activation of Vav/Rho GTPase signaling by CXCL12 controls membrane-type matrix metalloproteinase-dependent melanoma cell invasion. Cancer Res.

[29] Prieto-Sanchez RM, Hernandez JA, Garcia JL, Gutierrez NC, San Miguel J, Bustelo XR, Hernandez JM (2006). Overexpression of the VAV proto-oncogene product is associated with B-cell chronic lymphocytic leukaemia displaying loss on 13q. Br J Haematol.

[30] Dong Z, Liu Y, Lu S, Wang A, Lee K, Wang LH, Revelo M (2006). Vav3 oncogene is overexpressed and regulates cell growth and androgen receptor activity in human prostate cancer. Mol Endocrinol.

[31] Lyons LS, Burnstein KL (2006). Vav3, a Rho GTPase guanine nucleotide exchange factor, increases during progression to androgen independence in prostate cancer cells and potentiates androgen receptor transcriptional activity. Mol Endocrinol.

[32] Rao S, Lyons LS, Fahrenholtz CD, Wu F, Farooq A, Balkan W, Burnstein KL (2012). A novel nuclear role for the Vav3 nucleotide exchange factor in androgen receptor coactivation in prostate cancer. Oncogene.

[33] Chang KH, Sanchez-Aguilera A, Shen S, Sengupta A, Madhu MN, Ficker AM, Dunn SK, Kuenzi AM, Arnett JL, Santho RA, Agirre X, Perentesis JP, Deininger MW, Zheng Y, Bustelo XR, Williams DA (2012). Vav3 collaborates with p190-BCR-ABL in lymphoid progenitor leukemogenesis, proliferation, and survival. Blood.

[34] Citterio C, Menacho-Marquez M, Garcia-Escudero R, Larive RM, Barreiro O, Sanchez-Madrid F, Paramio JM, Bustelo XR (2012). The rho exchange factors vav2 and vav3 control a lung metastasis-specific transcriptional program in breast cancer cells. Sci Signal.

[35] Ahn J, Truesdell P, Meens J, Kadish C, Yang X, Boag AH, Craig AW (2013). Fer protein-tyrosine kinase promotes lung adenocarcinoma cell invasion and tumor metastasis. Mol Cancer Res.

[36] Havel LS, Kline ER, Salgueiro AM, Marcus AI (2015). Vimentin regulates lung cancer cell adhesion through a VAV2-Rac1 pathway to control focal adhesion kinase activity. Oncogene.

[37] Li HP, Huang HY, Lai YR, Huang JX, Chang KP, Hsueh C, Chang YS (2014). Silencing of miRNA-148a by hypermethylation activates the integrin-mediated signaling pathway in nasopharyngeal carcinoma. Oncotarget.

[38] He Z, Chen H, Li G, Zhu H, Gao Y, Zhang L, Sun J (2014). Diosgenin inhibits the migration of human breast cancer MDA-MB-231 cells by suppressing Vav2 activity. Phytomedicine.

[39] Menacho-Marquez M, Garcia-Escudero R, Ojeda V, Abad A, Delgado P, Costa C, Ruiz S, Alarcon B, Paramio JM, Bustelo XR (2013). The Rho exchange factors Vav2 and Vav3 favor skin tumor initiation and promotion by engaging extracellular signaling loops. PLoS Biol.

[40] Thalappilly S, Suliman M, Gayet O, Soubeyran P, Hermant A, Lecine P, Iovanna JL, Dusetti NJ (2008). Identification of multi-SH3 domain-containing protein interactome in pancreatic cancer: a yeast two-hybrid approach. Proteomics.

[41] Gyorffy B, Lanczky A, Eklund AC, Denkert C, Budczies J, Li Q, Szallasi Z (2010). An online survival analysis tool to rapidly assess the effect of 22,277 genes on breast cancer prognosis using microarray data of 1,809 patients. Breast Cancer Res Treat.

[42] Gyorffy B, Bottai G, Lehmann-Che J, Keri G, Orfi L, Iwamoto T, Desmedt C, Bianchini G, Turner NC, de The H, Andre F, Sotiriou C, Hortobagyi GN, Di Leo A, Pusztai L, Santarpia L (2014). TP53 mutation-correlated genes predict the risk of tumor relapse and identify MPS1 as a potential therapeutic kinase in TP53-mutated breast cancers. Mol Oncol.

[43] Mihaly Z, Kormos M, Lanczky A, Dank M, Budczies J, Szasz MA, Gyorffy B (2013). A meta-analysis of gene expression-based biomarkers predicting outcome after tamoxifen treatment in breast cancer. Breast Cancer Res Treat.

[44] Ferraro E, Peluso D, Via A, Ausiello G, Helmer-Citterich M (2007). SH3-Hunter: discovery of SH3 domain interaction sites in proteins. Nucleic Acids Res.

[45] Fabbiano S, Menacho-Marquez M, Sevilla MA, Albarran-Juarez J, Zheng Y, Offermanns S, Montero MJ, Bustelo XR (2014). Genetic dissection of the vav2-rac1 signaling axis in vascular smooth muscle cells. Mol Cell Biol.

[46] Yoshida T, Zhang Y, Rivera Rosado LA, Chen J, Khan T, Moon SY, Zhang B (2010). Blockade of Rac1 activity induces G1 cell cycle arrest or apoptosis in breast cancer cells through downregulation of cyclin D1, survivin, and X-linked inhibitor of apoptosis protein. Mol Cancer Ther.

[47] Montalvo-Ortiz BL, Castillo-Pichardo L, Hernandez E, Humphries-Bickley T, De la Mota-Peynado A, Cubano LA, Vlaar CP, Dharmawardhane S (2012). Characterization of EHop-016, novel small molecule inhibitor of Rac GTPase. J Biol Chem.

[48] Cornils H, Kohler RS, Hergovich A, Hemmings BA (2011). Downstream of human NDR kinases: impacting on c-myc and p21 protein stability to control cell cycle progression. Cell Cycle.

[49] Oehrl W, Kardinal C, Ruf S, Adermann K, Groffen J, Feng GS, Blenis J, Tan TH, Feller SM (1998). The germinal center kinase (GCK)-related protein kinases HPK1 and KHS are candidates for highly selective signal transducers of Crk family adapter proteins. Oncogene.

[50] Miller SL, DeMaria JE, Freier DO, Riegel AM, Clevenger CV (2005). Novel association of Vav2 and Nek3 modulates signaling through the human prolactin receptor. Mol Endocrinol.

[51] Alao JP (2007). The regulation of cyclin D1 degradation: roles in cancer development and the potential for therapeutic invention. Mol Cancer.

[52] Barbash O, Lin DI, Diehl JA (2007). SCF Fbx4/alphaB-crystallin cyclin D1 ubiquitin ligase: a license to destroy. Cell Div.

[53] Farago M, Dominguez I, Landesman-Bollag E, Xu X, Rosner A, Cardiff RD, Seldin DC (2005). Kinase-inactive glycogen synthase kinase 3beta promotes Wnt signaling and mammary tumorigenesis. Cancer Res.

[54] Li Q, Birkbak NJ, Gyorffy B, Szallasi Z, Eklund AC (2011). Jetset: selecting the optimal microarray probe set to represent a gene. BMC Bioinformatics.

[55] Rhodes DR, Kalyana-Sundaram S, Mahavisno V, Varambally R, Yu J, Briggs BB, Barrette TR, Anstet MJ, Kincead-Beal C, Kulkarni P, Varambally S, Ghosh D, Chinnaiyan AM (2007). Oncomine 3. 0: genes, pathways, and networks in a collection of 18,000 cancer gene expression profiles. Neoplasia.

[56] Edge SB, American Joint Committee on Cancer (2010). AJCC cancer staging manual.

[57] Lu TJ, Huang CY, Yuan CJ, Lee YC, Leu TH, Chang WC, Lu TL, Jeng WY, Lai MD (2005). Zinc ion acts as a cofactor for serine/threonine kinase MST3 and has a distinct role in autophosphorylation of MST3. J Inorg Biochem.

