# Supplementary material for: MST3 promotes proliferation and tumorigenicity through the VAV2/Rac1 signal axis in breast cancer
Source: Oncotarget. 2016 Feb 20;7(12):14586–604. doi: 10.18632/oncotarget.7542 (PMC4924737; doi:10.18632/oncotarget.7542)
Supplement: Supplementary file 1 [file oncotarget-07-14586-s001.pdf]

MST3 promotes proliferation and tumorigenicity through the VAV2/Rac1 signal axis in breast cancer

Supplementary Material

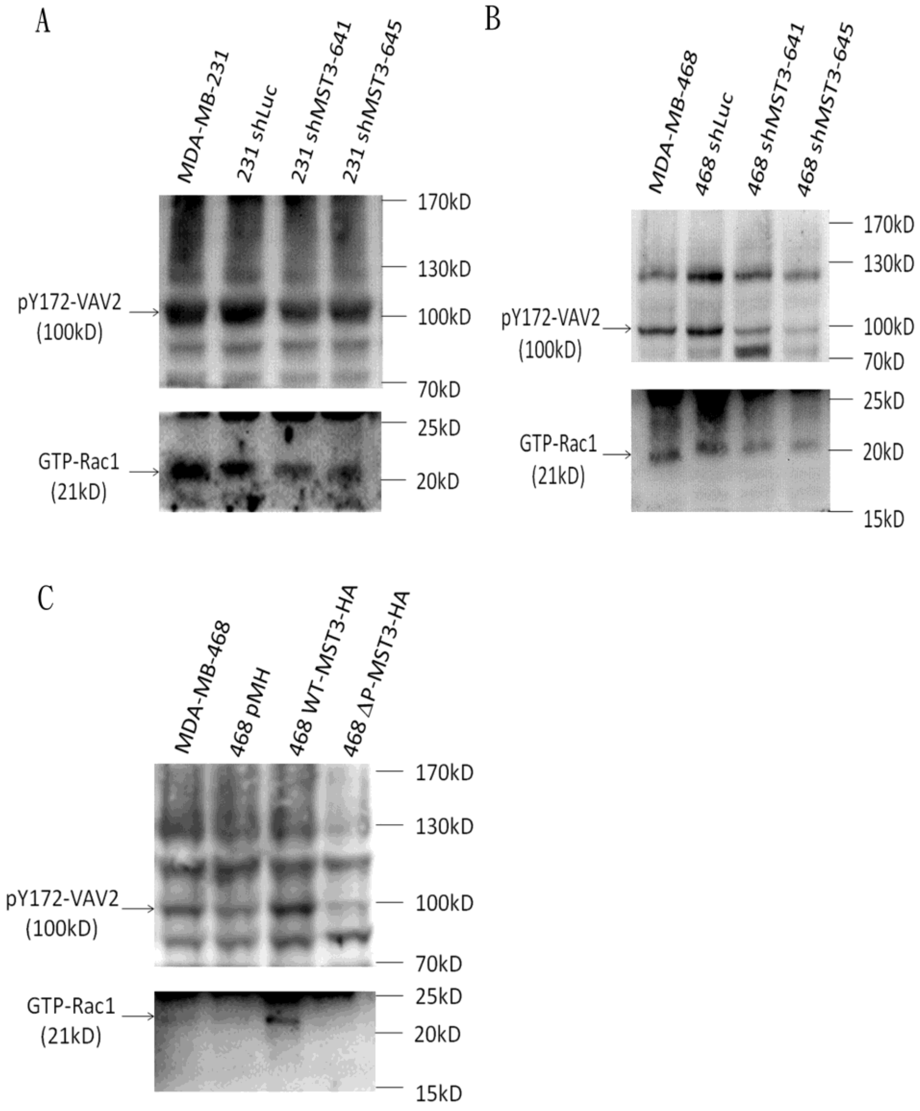

Supplementary Figure 1. The expression of phosphorylated VAV2 and GTP Rac1 in MDA-MB-231 and MDA-MB-468 stable transfectant cells by Western blotting. (A) Original images of pY172-VAV2 and GTP-Rac1 in figure 6A were shown. (B) Original images of pY172-VAV2 and GTP-Rac1 in figure 6C were shown. (C) Original images of pY172-VAV2 and GTP-Rac1 in figure 6E were shown.
